# Virulence determinants, drug resistance and mobile genetic elements of *Laribacter hongkongensis*: a genome-wide analysis

**DOI:** 10.1186/2045-3701-1-17

**Published:** 2011-04-19

**Authors:** Susanna KP Lau, Gilman KM Wong, Alan KL Tsang, Jade LL Teng, Rachel YY Fan, Herman Tse, Kwok-Yung Yuen, Patrick CY Woo

**Affiliations:** 1 State Key Laboratory of Emerging Infectious Diseases, Hong Kong; 2 Research Centre of Infection and Immunology, The University of Hong Kong, Hong Kong; 3 Carol Yu Centre of Infection, The University of Hong Kong, Hong Kong; 4 Department of Microbiology, The University of Hong Kong, Hong Kong

## Abstract

**Background:**

*Laribacter hongkongensis *is associated with community-acquired gastroenteritis and traveler's diarrhea. In this study, we performed an in-depth annotation of the genes in its genome related to the various steps in the infective process, drug resistance and mobile genetic elements.

**Results:**

For acid and bile resistance, *L. hongkongensis *possessed a urease gene cassette, two *arc *gene clusters and bile salt efflux systems. For intestinal colonization, it possessed a putative adhesin of the autotransporter family homologous to those of diffusely adherent *Escherichia coli *(*E. coli*) and enterotoxigenic *E. coli*. To evade from host defense, it possessed superoxide dismutase and catalases. For lipopolysaccharide biosynthesis, it possessed the same set of genes that encode enzymes for synthesizing lipid A, two Kdo units and heptose units as *E. coli*, but different genes for its symmetrical acylation pattern, and nine genes for polysaccharide side chains biosynthesis. It contained a number of CDSs that encode putative cell surface acting (RTX toxin and hemolysins) and intracellular cytotoxins (patatin-like proteins) and enzymes for invasion (outer membrane phospholipase A). It contained a broad variety of antibiotic resistance-related genes, including genes related to β-lactam (n = 10) and multidrug efflux (n = 54). It also contained eight prophages, 17 other phage-related CDSs and 26 CDSs for transposases.

**Conclusions:**

The *L. hongkongensis *genome possessed genes for acid and bile resistance, intestinal mucosa colonization, evasion of host defense and cytotoxicity and invasion. A broad variety of antibiotic resistance or multidrug resistance genes, a high number of prophages, other phage-related CDSs and CDSs for transposases, were also identified.

## Background

In 2001, *Laribacter hongkongensis*, a novel genus and species that belongs to the *Neisseriaceae *family of β-subclass of the Proteobacteria, was discovered from the blood and empyema pus of a patient with underlying alcoholic cirrhosis [1]. Subsequently, it was observed that *L. hongkongensis *was associated with freshwater fish borne community-acquired gastroenteritis and traveler's diarrhea in human [2-7]. The clinical syndrome of associated gastroenteritis is similar to those of *Salmonella *or *Campylobacter *gastroenteritis. About 80% and 20% of the patients have watery and bloody diarrhea respectively, one third of them have systemic symptoms and another one third have vomiting [4]. Pulsed-field gel electrophoresis of *Spe*I digested chromosomal DNA and multilocus sequence typing using seven housekeeping gene loci independently showed that the *L. hongkongensis *isolates recovered from freshwater fish and patients fell into separate clusters. These suggested that some *L. hongkongensis *clones could be more virulent or adapted to human than others [8,9].

For a gastrointestinal tract pathogen to cause infection, after transmission through the oral route, the bacterium has to be able to survive the hostile acidic environment of the stomach, resist the action of bile in the small intestine, colonize the gastrointestinal tract epithelium through binding of adhesins of the bacterium to receptors on epithelial cells, evade host immune defense mechanisms before causing diarrhea and/or invading the gastrointestinal tract and cause systemic infections, as in the case of bacteremia and empyema thoracis [1]. Moreover, the possession of drug resistance determinants and phages also enhance the potential capability of the bacterium to resist to killing by antimicrobials and causing diseases. In this article, we present an overview of the genes and gene cassettes of the *L. hongkongensis *genome related to these various steps in the infective process, as well as drug resistance and phages. The phylogeny of these genes, most of them were thought to be acquired through horizontal gene transfer, was also analyzed.

## Results and discussion

### Resistance to acid

#### Urease

Similar to other gastrointestinal tract pathogens, *L. hongkongensis *has to face the highly hostile and acidic environment of the stomach before reaching the intestine. *L. hongkongensis *possesses a urease, that is able to hydrolyze the limited amount of urea available in the stomach to generate carbon dioxide and ammonia, which increases the pH. In the *L. hongkongensis *genome, a complete urease cassette, that occupies a 7,556 bp region, is observed. The cassette includes eight CDSs, which encodes three urease structural proteins (UreA, UreB and UreC) and five accessory proteins (UreE, UreF, UreG, UreD and UreI) [10]. Similar to the urease of other bacteria, the urease of *L. hongkongensis *is presumably a nickel containing enzyme [11]. The histidine residues at the carboxyl terminal of UreE are supposed to bind to the nickel ions that are transported into *L. hongkongensis *through a nickel transporter, and donate the nickel ions to UreC during urease activation. Most of the eight genes in the urease cassette of *L. hongkongensis *are most closely related to their homologues in bacteria of α- and γ-proteobacteria, rather than those in other bacteria of β-proteobacteria [12-16].

#### Arginine deiminase

**Figure 1:**
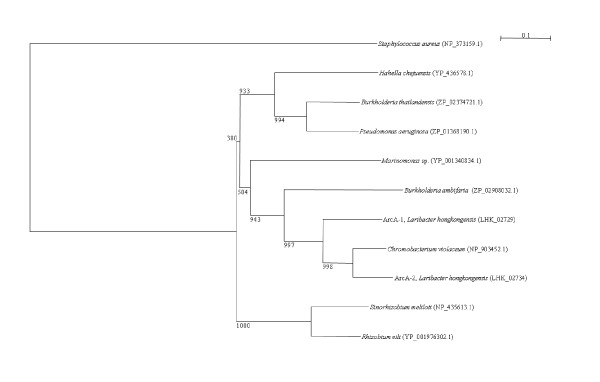
**Phylogenetic analysis of ArcA encoded in the *arc *gene cluster in *L. hongkongensis***. The tree was constructed by neighbor joining method using Kimura's correction and bootstrap values calculated from 1000 trees. Four hundred and nine and 409 amino acid positions in ArcA1 and ArcA2, respectively, were included in the analysis. The scale bars indicate the estimated number of substitutions per 10 amino acids. All names and accession numbers are given as cited in the GenBank database.

**Figure 2:**
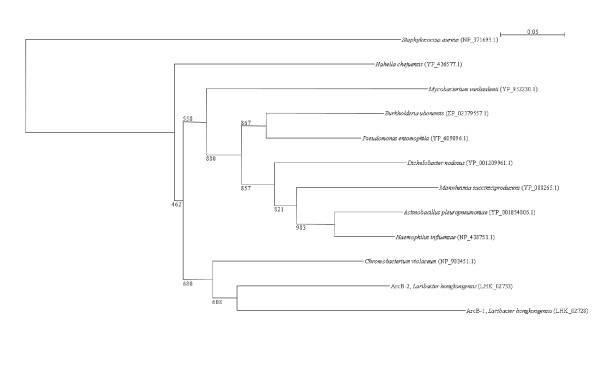
**Phylogenetic analysis of ArcB proteins encoded in the *arc *gene cluster in *L. hongkongensis***. The tree was constructed by neighbor joining method using Kimura's correction and bootstrap values calculated from 1000 trees. Three hundred and thirty-four and 335 amino acid positions in ArcB1 and ArcB2, respectively, were included in the analysis. The scale bar indicates the estimated number of substitutions per 20 amino acids. All names and accession numbers are given as cited in the GenBank database.

**Figure 3:**
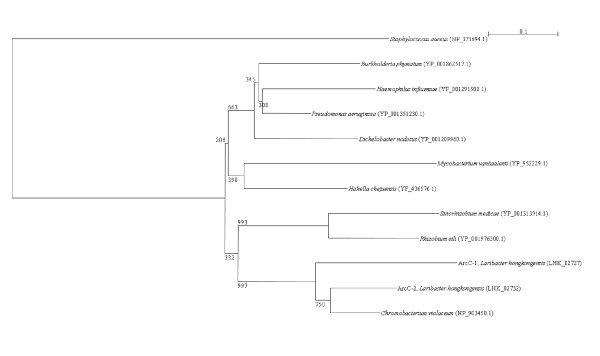
**Phylogenetic analysis of ArcC proteins encoded in the *arc *gene cluster in *L. hongkongensis***. The tree was constructed by neighbor joining method using Kimura's correction and bootstrap values calculated from 1000 trees. Two hundred and ninety-one and 314 amino acid positions in ArcC1 and ArcC2, respectively, were included in the analysis. The scale bars indicate the estimated number of substitutions per 10 amino acids. All names and accession numbers are given as cited in the GenBank database.

**Figure 4:**
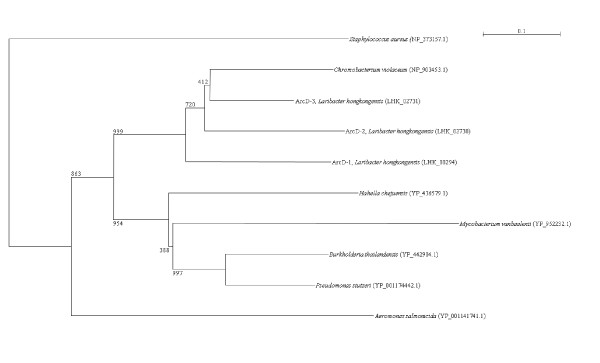
**Phylogenetic analysis of ArcD encoded in the *arc *gene cluster in *L. hongkongensis***. The tree was constructed by neighbor joining method using Kimura's correction and bootstrap values calculated from 1000 trees. Four hundred and ninety-two, 478 and 478 amino acid positions in ArcD1, ArcD2 and ArcD3, respectively, were included in the analysis. The scale bars indicate the estimated number of substitutions per 10 amino acids. All names and accession numbers are given as cited in the GenBank database.

**Figure 5:**
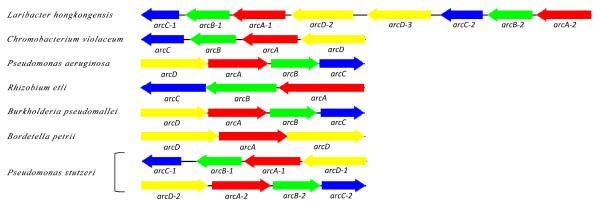
**Genetic organization of ADI clusters in *L. hongkongensis *and other representative microbial genomes**. The arrow boxes represent the CDSs. The relative positions of each gene are assigned as predicted by nucleotide sequence analysis.

Two *arc *gene clusters were encoded in the *L. hongkongensis *genome. Each cluster consists of four genes, *arcA*, *arcB*, *arcC *and *arcD*. *arcA*, *arcB *and *arcC *encode the three enzymes, arginine deiminase, ornithine carbamoyltransferase and carbamate kinase, of the arginine deiminase pathway, whereas *arcD *encodes a membrane bound arginine-ornithine antiporter. The arginine deiminase pathway converts L-arginine to carbon dioxide, ATP, and ammonia, which increases the pH. It has been shown in various bacteria, such as *Streptococcus sanguis*, *Streptococcus rattus*, *Streptococcus suis*, *Streptococcus pyogenes*, *Enterococcus faecium *and *Pseudomonas aeruginosa *that this gene cluster is useful for bacterial survival in acidic environment [17-19]. In *S. pyogenes*, it has also been shown that this pathway facilitates cell invasion and inhibits proliferation of human peripheral blood mononuclear cells [20,21]. Phylogenetically, these four genes of the *arc *gene cluster in *L. hongkongensis *are most closely related to the corresponding homologues in *Chromobacterium violaceum *(Figure 1, 2, 3, and 4), whereas the gene cluster is absent in *Neiserria meningitidis *and *Neisseria gonorrhoeae*. Among all bacteria with complete genomes sequenced, *L. hongkongensis *is the only one that contains two adjacent *arc *gene clusters (Figure 5).

### Bile resistance

#### Efflux pumps

Efflux of bile salts from bacteria is mediated through a number of efflux systems. These efflux systems pump a variety of compounds, including antibiotics, oxidative stress agents, organic solvents and bile salts, out of the bacterial cytoplasm. Among these efflux systems, the best studied one is encoded by *acrAB-tolC *of the resistance nodulation division (RND) family. This system has been shown to be present in the genomes of a variety of pathogenic and non-pathogenic bacteria of the human gastrointestinal tract, such as *Escherichia coli *and *Salmonella *Typhimurium [22,23]. In the *L. hongkongensis *genome, three complete copies of *acrAB-tolC*, of which AcrB is located in the inner membrane and contains the conserved ACR_tran domain, AcrA is located in the periplasmic space and contains the conserved HlyD domain and TolC as the outer membrane channel protein, are present. A recent bioinformatics analysis on bile resistance mechanisms in *Campylobacterales *also found that one complete copy of *acrAB-tolC *is present in the *C. jejuni *genome [24]. In addition to efflux pumps encoded by *acrAB-tolC*, the genome of *L. hongkongensis *also contains two copies of *emrAB-tolC *of the major facilitator superfamily, one copy of *acrAD-tolC *of the RND family (AcrD is also an inner membrane protein and contains the conserved ACR_tran domain similar to AcrB), one copy of *mdtABC*-*tolC *of the RND family and one copy of *ydgFE*/*mdtJI *of the small multidrug resistance family. These four gene cassettes were also found to be encoding efflux pumps related to bile resistance in *E. coli *[22,25-27]. In addition, *acrAD-tolC *and *mdtABC*-*tolC *have been documented to be related to bile salt resistance in *S*. Typhimurium [28].

#### Lipopolysaccharide (LPS) and Tol proteins

In addition to the efflux pumps, the integrity of the outer membrane is also important in resistance against bile. The O-antigen has been shown to be related to bile resistance in *S*. Typhimurium [29,30]. Tol proteins, which are cytoplasmic and periplasmic proteins encoded by a gene cluster that consists of five genes (*tolQ*, *tolR*, *tolA*, *tolB *and *pal*), are also important in maintaining the integrity of the outer membrane and bile resistance, as shown in *E. coli*, *S*. Typhimurium and *Erwinia chrysanthemi *[31-33]. In the genomes of *L. hongkongensis *and *C. violaceum*, *tolQ *was not clustered with *tolR*, *tolA*, *tolB *and *pal*, although all five genes are present in their genomes.

### Colonization of intestinal mucosa

**Figure 6:**
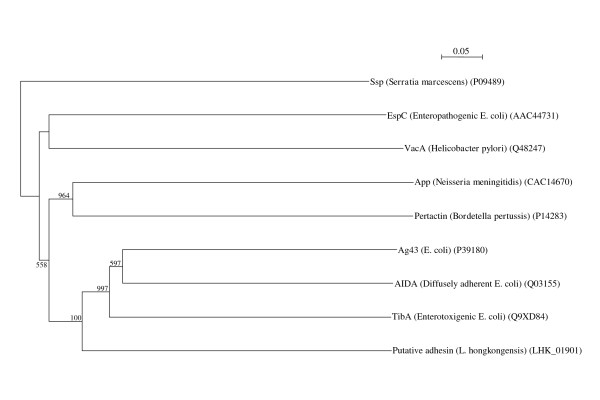
**Phylogenetic analysis of the putative adhesin of *L. hongkongensis***. The tree was constructed by neighbor joining method using Kimura's correction and bootstrap values calculated from 1000 trees. Six hundred and eight amino acid positions of the passenger domain were included in the analysis. The scale bar indicates the estimated number of substitutions per 20 amino acids. All names and accession numbers are given as cited in the GenBank database.

The first step of infection is adhesion to host cells. In the *L. hongkongensis *genome, a putative adhesin, with 27-30% amino acid identity to the adhesins of diffusely adherent *E. coli *(DAEC) [34-36] and enterotoxigenic *E. coli *(ETEC) [37-40], encoded by *aidA *and *tibA *respectively, was observed (Figure 6). It has been shown that *aidA *deletion mutants of DAEC lost the ability to adhere to HeLa cells and *tibA *deletion mutants of ETEC lost the ability to adhere to human intestine epithelial cells [37,41,42]; and *E. coli *HB101 transformed with *tib *loci was able to adhere to HCT 8 cells [37,42]. *aidA *and *tibA *encode proteins of the autotransporter family, type V protein secretion system of Gram-negative bacteria [43]. Proteins of this family possess three domains, an N-terminal signal sequence, a passenger or α-domain and a translocation or β-domain, which enable the proteins to transport themselves to cell surfaces. These three domains are all present in the putative adhesin in *L. hongkongensis*. Amino acid residues 1-36 is the putative signal sequence (predicted by SignalP). As in the passenger domains of other autotransporters, no cysteine residues, which were thought to interfere with transport of the proteins to cell surfaces because of formation of disulphide bonds, were present in the putative passenger domain (amino acid residues 37-756) of the putative adhesin in *L. hongkongensis *[41]. In the passenger domains of AIDA in DAEC, multiple copies of the consensus sequence VXNSGG, acceptor sites for heptose, addition of which catalyzed by AAH heptosyltransferase, encoded by *aah *located upstream to *aidA*, are present [44]. The addition of heptose was shown to be essential for the adhesion properties in the *tibA *adhesin in ETEC [45]. In the putative passenger domain of the putative adhesin in *L. hongkongensis*, nine copies of VXSGG, but not VXNSGG, were present; and a putative heptosyltransferase, with 52% amino acid identity to the TibC heptosyltransferase of ETEC, was present upstream to the putative adhesin gene in the *L. hongkongensis *genome. Interestingly, in the putative passenger domain of *tibA *adhesin in ETEC, 11 copies of VXSGG, but not VXNSGG, were present, but whether VXSGG is the acceptor sites for heptose has not been documented. In addition to their roles for adhesion, the passenger domains may also possess virulence functions, such as autoaggregation, biofilm formation, invasion and cytotoxicity. In the putative translocation domain, the consensus motif (Y/V/I/F/W)-X-(F/W) at the extreme carboxyl terminus of other autotransporter proteins, predicted to play a role in outer membrane localization and/or stability of these proteins, was present [41].

### Evasion of host defense

To protect from the active oxygen species (superoxide and hydrogen peroxide) released from phagocytic cells, the genome of *L. hongkongensis *encodes superoxide dismutase and catalases, in line with its catalase-positive phenotype. The putative superoxide dismutase of *L. hongkongensis*, which decomposes superoxide to hydrogen peroxide and oxygen, is most closely related to those of *C. violaceum*, *N. meningitidis *and *N. gonorrhoeae*. There are three putative catalases in the *L. hongkongensis *genome, encoded by a *katE *(encoding hydroperoxidase II) and two *katG *(encoding hydroperoxidase I with catalase-peroxidase activity). These decompose hydrogen peroxide to water and oxygen. *katE *in *L. hongkongensis *is most closely related to the homologues in *Ralstonia eutropha*, whereas the two *katG *were most closely related to those in *Shewanella amazonensis *and *Vibrio cholerae *respectively. In addition to protection against the active oxygen species, some efflux pumps may export host-derived antimicrobial agents in addition to antibiotics, bile and other substances, hence protecting from such naturally produced molecules of the host.

### Virulence factors

#### Lipopolysaccharide

LPS consists of three parts: lipid A, core oligosaccharide, and polysaccharide side chains. In *E. coli*, the minimal LPS required for growth include lipid A and two keto-deoxyoctulonate (Kdo) units of the core oligosaccharide. The LPS of wild type strains of *E. coli *consist of additional core sugars and polysaccharide side chains. The polysaccharide side chains are also known as the O-antigen, which varies among different species of Gram-negative bacteria and different strains of the same species. These sugars enhance survival during environmental stress, and help the bacteria evade the host immune system by modification of the structure. Lipid A, also known as the endotoxin, is the hydrophobic anchor of LPS. It is a glucosamine based phospholipid inserted into the outer membranes of most Gram-negative bacteria. Most Gram-negative bacteria synthesize lipid A by pathways similar to the one in *E. coli*. Through binding to Toll-like receptor 4 and CD14, lipid A of Gram-negative bacteria trigger the synthesis and secretion of pro-inflammatory cytokines. The actions of these cytokines lead to local and systemic inflammatory responses, which result in various clinical manifestations, and even deaths, of patients.

**Figure 7:**
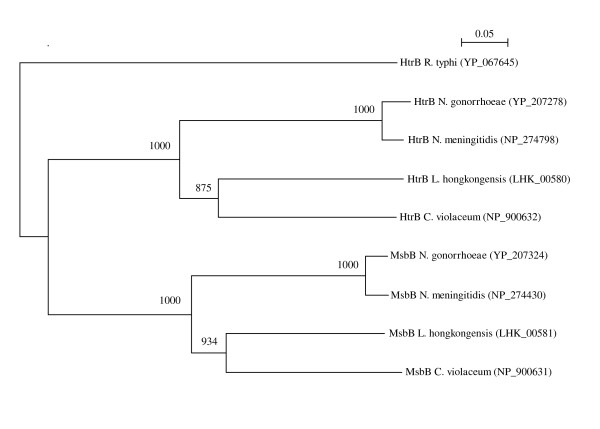
**Phylogenetic analysis of confirmed/putative HtrB and MsbB of *L. hongkongensis*, *C. violaceum*, *N. meningitidis *and *N. gonorrhoeae***. The tree was constructed by neighbor joining method using Kimura's correction and was rooted using HtrB of *Rickettsia typhi *(YP_067645). Two hundred and eighty-four amino acid positions were included in the analysis. The scale bar indicates the estimated number of substitutions per 20 amino acids. Numbers at nodes indicated levels of bootstrap support calculated from 1000 trees. All names and accession numbers are given as cited in the GenBank database.

The same set of genes that encode enzymes in the biosynthetic pathways of lipid A, the two Kdo units and the heptose units are present in the *L. hongkongensis*, *C. violaceum*, *N. meningitidis*, *N. gonorrhoeae *and *E. coli *genomes. In contrast to *E. coli*, the lipid A of *C. violaceum*, *N. meningitidis *and *N. gonorrhoeae *had a symmetrical acylation pattern [46]. Both the reducing and terminal N-acetyl-glucosamine residues in these bacteria carry three acyl groups. The sequential addition of the last 12-carbon acyl group to the reducing and terminal N-acetyl-glucosamine residues are catalyzed by enzymes encoded by the *htrB *and *msbB *genes, respectively. It was found that *msbB *deletion mutants of *N. meningitidis *and *N. gonorrhoeae *had lower abilities to activate human macrophages to produce pro-inflammatory cytokines [47-49]. Phylogenetic analysis of the experimentally confirmed *htrB *and *msbB *genes in *N. meningitidis *and *N. gonorrhoeae *and the putative *htrB *and *msbB *genes in *L. hongkongensis *and *C. violaceum *showed that the four *htrB *genes and the four *msbB *genes fell into two separate clusters, with very high bootstrap values (Figure 7). Therefore, we speculate that the *htrB *and *msbB *genes in *L. hongkongensis *and *C. violaceum *serve similar functions as those in *N. meningitidis *and *N. gonorrhoeae *and that the lipid A of *L. hongkongensis *also had a symmetrical acylation pattern.

The genes that are responsible for the synthesis of α-chain L1, α-chain L2, β-chain and γ-chain in the core oligosaccharide in *N. meningitidis *and *N. gonorrhoeae *(*lgtA, lgtB, lgtC, lgtD, lgtE, lgtF, lgtG, rfaK*) and those for the addition of sialic acids to these chains (*lst*) are absent in the genomes of *L. hongkongensis *and *C. violaceum *[50]. On the other hand, nine genes which encode putative enzymes for biosynthesis of the polysaccharide side chains are present in the *L. hongkongensis *genome. Four of these genes (*rfbA*, *rfbB*, *rfbC *and *rfbD*) are also present in the genomes of *C. violaceum*, *N. meningitidis *and *N. gonorrhoeae*. The enzymes encoded by these four genes catalyzed reactions for the synthesis of dTDP-rhamnose, although mutations of them in *N. meningitidis *and *N. gonorrhoeae *did not result in any change in their phenotypes [51,52]. The other five genes (*wbmF*, *wbmG*, *wbmH*, *wbmI *and *wbmK*), which encode putative nucleotide sugar epimerases/dehydratases and amidotransferase, are not present in the *C. violaceum*, *N. meningitidis *and *N. gonorrhoeae *genomes, but are most closely related to the corresponding genes for the biosynthesis of the O-antigens in *Bordetella parapertussis *and *Bordetella bronchoseptica *[53]. Although the structures of the LPS of *L. hongkongensis *and *C. violaceum *remain to be determined, these imply that the structures of the LPS of *L. hongkongensis *and *C. violaceum *are probably quite different from those of the lipooligosaccharides of *N. meningitidis *and *N. gonorrhoeae*.

Recently, a number of genes that encode proteins for the assembly and transport of LPS in *E. coli *have been discovered [54]. All these genes were also present in the genomes of *L. hongkongensis*, *C. violaceum*, *N. meningitidis *and *N. gonorrhoeae *(Table 1). The exact functions of these proteins have not been fully elucidated.

**Table 1:** Genes for assembly and transport of LPS

Protein	Gene	*E. coli *str. K-12 substr. MG1655	*L. hongkongensis*	*C. violaceum*	*N. gonorrhoeae *FA1090	*N. meningitidis *MC58
Periplasmic LPS-binding protein	*lptA*	b3200	LHK_02023	CV3330	NGO1606	NMB0355
Lipopolysaccharide export, IM-tethered periplasmic protein of LptBFGC export complex	*lptC*	b3199	LHK_02022	CV3329	NGO1607	NMB0354
Lipopolysaccharide export ABC transporter ATP-binding protein of LptBFGC export complex	*lptB*	b3201	LHK_02024	CV3331	NGO1605	NMB0356
Lipopolysaccharide export ABC permease of LptBFGC export complex	*lptF*	b4261	LHK_01413	CV2915	NGO1228	NMB1570
Lipopolysaccharide export ABC permease of LptBFGC export complex	*lptG*	b4262	LHK_01412	CV2916	NGO1229	NMB1571
LPS assembly OM complex LptDE, beta-barrel component	*lptD*	b0054	LHK_03193	CV4229	NGO1715	NMB0280
LPS assembly OM complex LptDE, lipoprotein component	*lptE*	b0641	LHK_00118	CV0506	NGO0282	NMB0707

#### Cytotoxins

The *L. hongkongensis *genome contains a number of CDSs that encode putative cytotoxins. These include cell surface acting cytotoxins, such as RTX toxin and hemolysins; and intracellular cytotoxins such as patatin-like proteins.

##### RTX toxins

**Figure 8:**
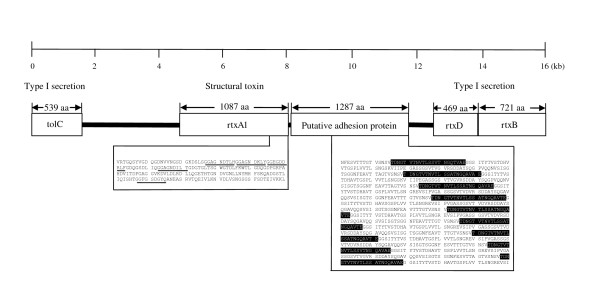
**Genetic organization of the RTX gene cluster (*tolC-rtxA1-rtxD-rtxB*) in *L. hongkongensis***. The boxes represent the CDSs. The number of amino acid residues of each gene is indicated above the boxes. The basic functional activities of the corresponding gene products are given on the top. Five copies of glycine-rich nonapeptide repeats (GGXGXDX[L/I/V/W/Y/F]X, where X is any amino acid) of *rtxA1 *are underlined. An CDS of unknown function, located between *rtxA1 *and *rtxD*, are also depicted, where nine repeats of 22 amino acids are highlighted. The relative positions of each gene are assigned as predicted by nucleotide sequence analysis.

**Figure 9:**
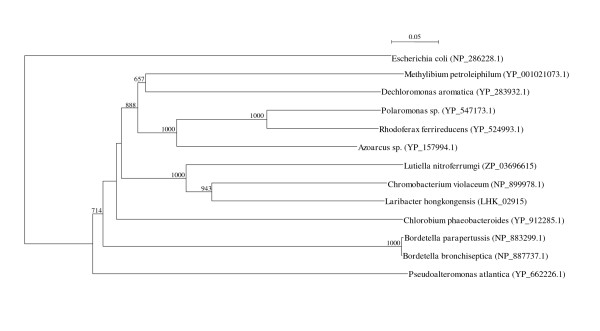
**Phylogenetic analysis of TolC in the RTX gene cluster of *L. hongkongensis***. The tree was constructed by neighbor joining method using Kimura's correction and bootstrap values calculated from 1000 trees. Four hundred and forty-two amino acid positions were included in the analysis. The scale bars indicate the estimated number of substitutions per 20 amino acids. All names and accession numbers are given as cited in the GenBank database.

**Figure 10:**
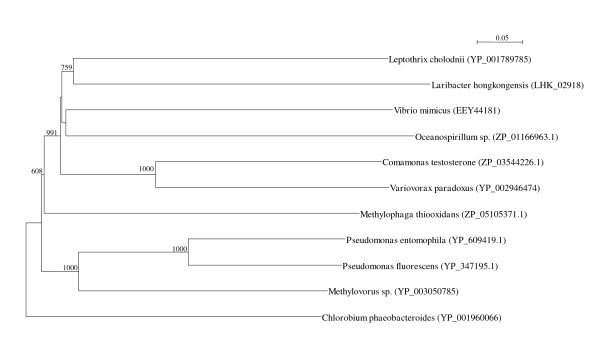
**Phylogenetic analysis of RtxA1 in the RTX gene cluster of *L. hongkongensis***. The tree was constructed by neighbor joining method using Kimura's correction and bootstrap values calculated from 1000 trees. One thousand and eighty-seven amino acid positions were included in the analysis. The scale bars indicate the estimated number of substitutions per 20 amino acids. All names and accession numbers are given as cited in the GenBank database.

**Figure 11:**
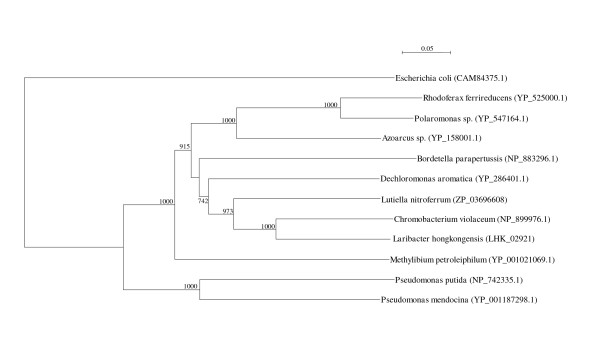
**Phylogenetic analysis of RtxD in the RTX gene cluster of *L. hongkongensis***. The tree was constructed by neighbor joining method using Kimura's correction and bootstrap values calculated from 1000 trees. Four hundred and fifty-two amino acid positions were included in the analysis. The scale bars indicate the estimated number of substitutions per 20 amino acids. All names and accession numbers are given as cited in the GenBank database.

**Figure 12:**
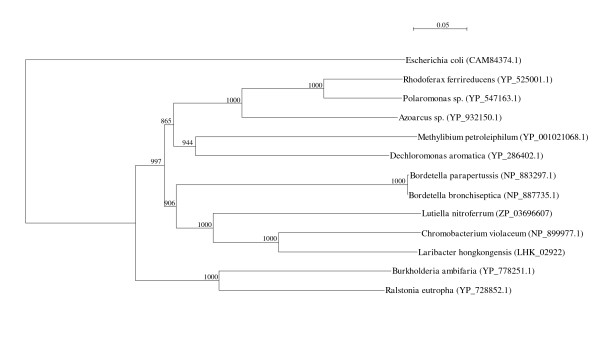
**Phylogenetic analysis of RtxB in the RTX gene cluster of *L. hongkongensis***. The tree was constructed by neighbor joining method using Kimura's correction and bootstrap values calculated from 1000 trees. Seven hundred and twenty amino acid positions were included in the analysis. The scale bars indicate the estimated number of substitutions per 20 amino acids. All names and accession numbers are given as cited in the GenBank database.

RTX toxins, originally discovered in *E. coli *(α-hemolysin) [55,56], are most commonly found in bacteria of the *Pasteurellaceae *family. Most RTX toxins are hemolysins or leukotoxins [57,58]. The *L. hongkongensis *genome contains an RTX gene cluster (*tolC*-*rtxA1*-*rtxD*-*rtxB*) and an isolated *rtxA2 *gene. In the RTX gene cluster (Figure 8), *tolC *encodes the outer membrane component of the type I secretion apparatus, *rtxA1 *encodes the structural toxin, *rtxD *encodes the adaptor protein anchored to the inner membrane and *rtxB *encodes the inner membrane ATPase. TolC, RtxD and RtxB form the secretion apparatus for exporting RtxA. Similar to RtxA of other bacteria, RtxA1 and RtxA2 of *L. hongkongensis *possess tandem arrays of glycine-rich nonapeptide repeats (GGXGXDX[L/I/V/W/Y/F]X, where X is any amino acid) for binding of calcium ions (Figure 8). There are five nonapeptide repeats in RtxA1 and nine nonapeptide repeats in RtxA2. Unlike most other bacteria which contain *rtxC *genes, the RTX gene cluster of *L. hongkongensis *does not possess this gene. Instead, it contains a gene of putative adhesive function, located between *rtxA1 *and *rtxD*. Domain search using InterProScan showed that this gene contains nine repeats of 22 amino acids (TDNGTVTNVTLSSVTNGQTVAE) with parallel beta-helix structures. Each repeat is separated from the adjacent one by 82 amino acids (Figure 8). Although the genomes of *L. hongkongensis*, *C. violaceum *and *N. meningitidis *all contain RTX toxin, RtxA1 and RtxA2 of *L. hongkongensis *do not show clustering with the homologues in *C. violaceum *and *N. meningitidis*. This is in contrast to the other genes (*tolC*, *rtxD *and *rtxB*) in the RTX gene cluster, which are all most closely related to the corresponding homologues in *C. violaceum *and other species of β-proteobacteria [59,60] (Figure 9, 10, 11, and 12). Moreover, the amino acid identities between TolC, RtxD and RtxB and their homologues in *C. violaceum *are much higher than those between RtxA1 or RtxA2 and their homologues in any other bacteria (Figure 9, 10, 11, and 12). These suggest that *rtxA1 *and *rtxA2 *have evolved much faster than *tolC*, *rtxD *and *rtxB*, so that the toxins can bind to their corresponding host cells more efficiently. Interestingly, similar to *rtxA2 *of *L. hongkongensis*, the structural toxin genes (*frpC *and *frpA*) in *N. meningitidis *are not linked to genes of the type I secretion system. However, it has been shown that FrpC and FrpA can be secreted by *E. coli *harboring *hlyBD *genes, indicating that they are probably secreted by secretion systems unlinked to their corresponding genes [61].

##### Hemolysins

In the *L. hongkongensis *genome, there are two gene loci that encode putative hemolysins. The first putative hemolysin contains three domains, the first one of the DUF21 superfamily, the second one of the CBS_pair superfamily and the third one of the CorC_HlyC superfamily. Among the five most closely related protein sequences, three of them were putative hemolysins of three different *Yersinia *species, and the other two were hypothetical proteins. The second putative hemolysin belongs to the HlyIII superfamily, which contains seven transmembrane domains with conserved amino acid residues present. It is most closely related to the hemolysin III of *C. violaceum*.

##### Patatin-like protein

**Figure 13:**
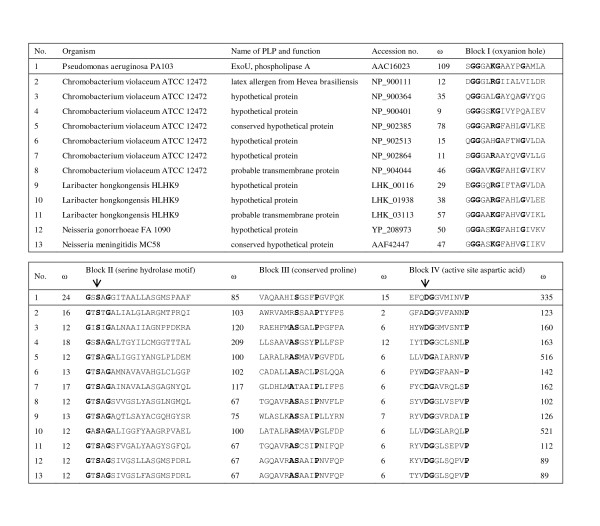
**Multiple alignments of the four conserved domains in the putative patatin-like proteins in the genomes of *L. hongkongensis*, *C. violaceum*, *N. meningitidis *and *N. gonorrhoeae***. The two arrows indicate the Ser-Asp catalytic dyad. Conserved amino acids in the four domains are in bold. ω, number of amino acids before and after the conserved domains.

Patatin, originally described in plants such as potatoes, has diverse functions such as storage glycoproteins [62], signal transduction [63] and defense against parasites [64]. In 2003, it was found that toxin ExoU of *P. aeruginosa*, delivered to eukaryotic cells via a type III secretion system, possessed the catalytic domains of patatin, iPLA(2) and cPLA(2) [65]. Direct injection of ExoU in mammalian cells resulted in irreversible damage to cellular membranes and rapid necrotic death [66]. Similar to patatin, ExoU of *P. aeruginosa *possessed phopholipase A2 activity. *P. aeruginosa *mutants with mutations at the active sites of the patatin-like protein were less virulent than wild type *P. aeruginosa *in a mouse model [67]. Subsequently, genes that encode putative patatin-like proteins were observed in many bacterial genomes, although none of them was characterized phenotypically [68]. It was also observed that the average copy number of genes that encode patatin-like proteins is higher in plant/animal bacterial pathogens than in non-pathogens [68]. In some pathogens, up to eight copies of genes that encode putative patatin-like proteins can be found. Similar to *P. aeruginosa*, the genome of *L. hongkongensis *also contains three copies of genes that encode putative patatin-like proteins. The lengths of the genes that encode putative patatin-like proteins in the genomes of *L. hongkongensis*, *C. violaceum *(7 copies), *N. meningitidis *(1 copy) and *N. gonorrhoeae *(1 copy) varied from 894 to 2,337 bp. The three copies in the *L. hongkongensis *genome are 951, 963 and 2,232 bp respectively. All three copies contain all the four domains that can be found in bacterial patatin-like proteins, including a putative oxyanion hole, a serine hydrolase G-X-S-X-G domain, a potential serine-containing phosphorylation site and an aspartate-containing active site domain (Figure 13). The serine in the hydrolase domain and the aspartate made up a patatin-specific catalytic dyad that has not been described in any other known proteins [68].

#### Enzymes

##### Outer membrane phospholipase A

**Figure 14:**
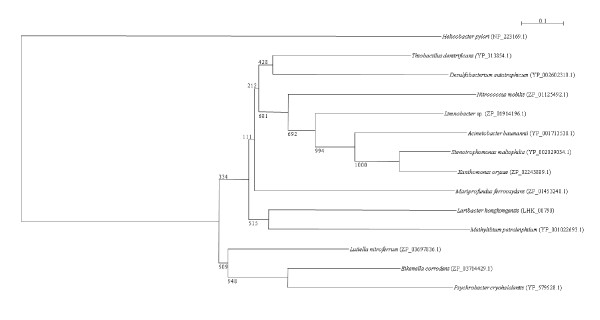
**Phylogenetic analysis of outer membrane phospholipase A of *L. hongkongensis***. The tree was constructed by neighbor joining method using Kimura's correction and bootstrap values calculated from 1000 trees. Three hundred and seventy-seven amino acid positions were included in the analysis. The scale bar indicates the estimated number of substitutions per 10 amino acids. All names and accession numbers are given as cited in the GenBank database.

It has been shown that outer membrane phospholipase A (OMPLA) is a virulence factor in a number of bacteria, including *Helicobacter pylori *and *C. coli*. Located on the outer membrane of bacteria, OMPLA lyses the outer membrane, leading to release of other virulence factors, such as urease and VacA in *H. pylori*. In the *L. hongkongensis *genome, a gene that encodes a putative OMPLA is observed. This OMPLA possesses a complete and highly specific consensus sequence motif (YTQ-X_n_-G-X_2_-H-X-SNG) found in OMPLA of other bacteria. Phylogenetically, it is most closely related to the OMPLA of *Methylibium petroleiphilum*, a methyl tert-butyl ether-degrading methylotroph of β-proteobacteria (Figure 14) [69].

### Drug resistance

A genome-wide analysis using similarity searches revealed the presence of a large number of antibiotic resistance-related genes in *L. hongkongensis *strain HLHK9. They are related to β-lactam (Table 2), multidrug efflux (Table 3) and other resistance genes (Table 4).

**Table 2:** CDSs related to beta-lactam antibiotics in *L. hongkongensis*

CDS	Gene	Product	Organism with the closest matching sequences	E-value	Identities	Remarks^a^
LHK_00876		β-lactamase domain protein	*Thauera *sp.	6e-77	135/204 (66%)	
LHK_00878	*gloB *	Hydroxyacylglutathione hydrolase	*Rickettsiella grylli*	6e-64	126/259 (48%)	
LHK_00975	*dacC*	D-alanyl-D-alanine-carboxypeptidase	*C. violaceum*	e-140	254/379 (67%)	PBP6a
LHK_02726	*pbpG*	D-alanyl-D-alanine-endopeptidase	*C. violaceum*	4e-94	183/288 (63%)	PBP7
LHK_02764	*prc*	Carboxy-terminal processing protease	*C. violaceum*	1e-173	315/480 (65%)	PBP3 processing protease
LHK_02836	*dacB*	Serine-type D-Ala-D-Ala carboxypeptidase	*C. violaceum*	3e-81	207/427 (48%)	PBP4a
LHK_02959	*mrcA*	Peptidoglycan glycosyltransferase	*C. violaceum*	0	512/795 (64%)	PBP1a
LHK_03028	*ampC*	β-lactamase	*C. violaceum*	7e-91	189/381 (49%)	
LHK_03062	*ftsI*	Penicillin-binding protein 3 precursor	*C. violaceum*	0	349/586 (59%)	PBP3
LHK_03073	*mrdA*	Penicillin-binding protein 2	*C. violaceum*	0	404/583 (69%)	PBP2

^a^PBP, penicillin-binding protein

**Table 3:** CDSs related to multidrug resistance in *L. hongkongensis*

CDS	Gene	Product	Organism with the closest matching sequence	E-value	Identities	No. of TMS^a^	Remarks^b^
LHK_00138	*tolC*	TolC family type I secretion outer membrane protein	*Polaromonas naphthalenivorans*	2e-72	203/455 (44%)	--	OMP
LHK_00140	*acrB*	Acriflavin resistance protein	*P. naphthalenivorans*	0	723/1066 (67%)	13	RND
LHK_00141	*acrA*	Efflux transporter, RND family, MFP subunit	*P. naphthalenivorans*	2e-51	153/355 (43%)	--	MFP
LHK_00142	*arsR*	Transcription regulator ArsR	*Bordetella parapertussis*	2e-26	62/99 (62%)	--	TR
LHK_00221		RND efflux system outer membrane lipoprotein	*Pelobacter propionicus*	1e-108	207/424 (48%)	--	OMP
LHK_00222	*macB*	Macrolide-specific ABC-type efflux carrier	*Bordetella avium*	0	429/655 (65%)	4	ABC
LHK_00223	*macA*	Efflux transporter, RND family, MFP subunit	*Lutiella nitroferrum*	4e-127	252/384 (65%)	--	MFP
LHK_00466		Hypothetical protein	*Dorea longicatena*	6e-45	131/439 (29%)	12	MATE
LHK_00743	*mdfA*	Probable multidrug translocase protein	*C. violaceum*	e-139	253/394 (64%)	12	MFS
LHK_01214		Probable multiple antibiotic resistance protein MarC	*C. violaceum*	7e-58	118/208 (56%)	--	MarC
LHK_01285	*mdtA*	Probable membrane protein	*C. violaceum*	3e-94	188/340 (55%)	--	MFP
LHK_01286_01287	*mdtB*	Drug efflux pump transmembrane protein	*C. violaceum *	0	700/1018 (68%)	12	RND
LHK_01288	*mdtC*	Drug efflux pump transmembrane protein	*C. violaceum*	0	678/994 (68%)	10	RND
LHK_01289	*tolC*	Putative outer membrane protein precursor	*Acinetobacter sp*.	4e-79	189/433 (43%)	--	OMP
LHK_01373	*emrB*	Multidrug resistance protein	*C. violaceum*	0	323/490 (65%)	14	MFS
LHK_01374	*emrA*	Multidrug efflux membrane fusion protein	*Ralstonia eutropha*	e-101	194/370 (52%)	--	MFP
LHK_01375	*mdtP*	Outer membrane efflux protein	*Yersinia enterocolitica*	1e-38	142/469 (30%)	--	OMP
LHK_01376	*emrR*	MarR family transcriptional regulator	*C. violaceum*	4e-32	69/156 (44%)	--	TR
LHK_01383		Probable multiple antibiotic resistance protein MarC	*C. violaceum*	4e-80	149/232 (64%)	--	MarC
LHK_01384	*mdtJ*	Multidrug efflux system protein MdtJ	*Klebsiella pneumoniae*	3e-20	52/119 (43%)	3	SMR
LHK_01385	*mdtI*	Multidrug efflux system protein MdtI	*Salmonella enterica*	6e-21	63/109 (57%)	4	SMR
LHK_01424		RND efflux system, outer membrane lipoprotein, NodT family	*Syntrophobacter fumaroxidans*	7e-108	223/446 (50%)	--	OMP
LHK_01425		Transporter, hydrophobe/amphiphile efflux-1 (HAE1) family	*Pelobacter propionicus*	0	553/1036 (53%)	12	RND
LHK_01426		Efflux transporter, RND family, MFP subunit	*S. fumaroxidans*	7e-95	186/364 (51%)	--	MFP
LHK_01870		Putative multidrug resistance protein	*R. eutropha*	8e-47	153/483 (31%)	14	MFS
LHK_01934		Probable multiple antibiotic resistance protein MarC	*C. violaceum*	3e-80	152/205 (74%)	--	MarC
LHK_01967		ABC transporter, transmembrane region:ABC transporter related	*R. eutropha*	0	550/732 (75%)	6	ABC
LHK_02051		Lipoprotein releasing system, ATP-binding protein	*Pseudomonas stutzeri*	4e-58	134/227 (59%)	--	ABC
LHK_02129	*mexA*	Multidrug resistance protein	*Xanthomonas campestris*	3e-104	223/375 (59%)	--	MFP
LHK_02130	*acrB*	AcrB/AcrD/AcrF family protein	*Cellvibrio japonicus*	0	768/1034 (74%)	14	RND
LHK_02131	*nodT*	RND efflux system, outer membrane lipoprotein, NodT	*Geobacter metallireducens*	8e-142	266/466 (57%)	--	OMP
LHK_02132		Transcriptional regulator, TetR/AcrR family	*Cellvibrio japonicus*	2e-46	93/187 (49%)	--	TR
LHK_02173		Probable MFS transporter	*C. violaceum*	1e-82	195/370 (52%)	12	MFS
LHK_02235		Putative integral membrane efflux protein	*Yersinia pestis*	0	379/505 (75%)	13	abgT family protein
LHK_02238		ABC transporter	*Azoarcus sp*.	e-157	281/371 (75%)	7	ABC
LHK_02239	*yhiH*	ABC transporter related	*Thauera sp*.	0	699/954 (73%)	6	ABC
LHK_02240		Conserved hypothetical protein, predicted secretion protein HlyD family	*Azoarcus sp*.	6e-96	232/339 (68%)	--	MFP
LHK_02241	*oprM3*	Outer membrane efflux protein	*B. avium*	1e-100	241/453 (53%)	--	OMP
LHK_02292		Probable multiple antibiotic resistance protein MarC	*C. violaceum*	2e-60	116/218 (53%)	--	MarC
LHK_02533		Multidrug efflux protein NorA	*C. violaceum*	e-122	230/447 (51%)	12	MATE
LHK_02539		EmrB/QacA family drug resistance transporter	*P. stutzeri*	2e-147	277/481 (57%)	13	MFS
LHK_02783		Hypothetical protein multiple antibiotic resistance (MarC)-related protein	*C. violaceum*	5e-74	139/200 (69%)	--	MarC
LHK_02825	*natC*	Periplasmic type I secretion system	*C. violaceum*	4e-82	190/439 (43%)	--	OMP
LHK_02826	*acrB*	Probable transmembrane drug efflux protein	*C. violaceum*	0	693/1019 (68%)	12	RND
LHK_02827	*acrA*	Probable transport/efflux transmembrane protein	*C. violaceum*	3e-83	174/351 (49%)	--	MFP
LHK_02828	*acrR*	TetR/AcrR family transcriptional regulator	*C. violaceum*	6e-44	92/183 (50%)	--	TR
LHK_02929	*acrA*	Probable multidrug efflux membrane permease	*C. violaceum*	1e-89	203/372 (54%)	--	MFP
LHK_02930	*acrD*	Acriflavin resistance protein D	*C. violaceum*	0	717/1036 (69%)	12	RND
LHK_02931	*oprM*	Outer membrane efflux protein	*C. violaceum*	e-136	252/467 (53%)	--	OMP
LHK_02949	*msbA*	Transport ATP-binding protein MsbA	*C. violaceum*	0	344/554 (62%)	5	ABC
LHK_02975	*bcr*	Probable MFS transporter	*C. violaceum*	e-147	269/388 (69%)	12	MFS
LHK_03132	*emrB*	Probable multidrug resistance protein	*C. violaceum*	0	303/492 (61%)	14	MFS
LHK_03133	*emrA*	Multidrug resistance protein	*Burkholderia thailandensis*	1e-108	202/377 (53%)	--	MFP
LHK_03134	*tolC*	Outer membrane efflux protein	*R. eutropha*	6e-45	153/453 (33%)	--	OMP

*^a^*TMS, transmembrane segment domain

^b^RND, resistance-nodulation-division family; MFS, major facilitator superfamily; ABC, ATP-binding cassette transporter superfamily; SMR, small multidrug resistance family; MATE, multidrug and toxic compound extrusion; MFP, membrane fusion protein; OMP, outer membrane (channel) protein; TR, transcription regulator; MarC, MarC-like protein.

**Table 4:** Miscellaneous resistance genes in *L. hongkongensis*

CDS	Gene	Product	Organism with the closest matching sequence	E-value	Identities
LHK_00025	*ksgA*	Dimethyladenosine transferase	*C. violaceum*	1e-94	178/260 (68%)
LHK_00913		Arsenical-resistance protein	*Burkholderia oklahomensis*	2e-65	153/183 (83%)
LHK_01038	*crcB*	Camphor resistance protein CrcB	*Brucella abortus*	2e-33	88/129 (68%)
LHK_01039	*crcB*	Camphor resistance protein CrcB	*Y. pestis*	3e-34	78/123 (63%)
LHK_01350	*rarD*	RarD protein, chloamphenicol sensitive	*C. violaceum*	2e-81	172/285 (60%)
LHK_02940	*bacA*	Undecaprenol kinase, putative bacitracin resistance protein	*Burkholderia graminis*	3e-83	174/278 (62%)

#### β-lactam resistance-related genes

A total of 10 CDSs related to β-lactam resistance were identified in the *L. hongkongensis *genome. Genes that exhibit similarity to penicillin-binding proteins (PBPs) (6 CDSs) of other bacterial species were found (Table 2). The PBPs identified in *L. hongkongensis *include PBP1a, PBP2, PBP3, PBP4a, PBP6a, and PBP7, which are essential proteins that are involved in biosynthesis of murein and peptidoglycan, and are targets for inhibition by β-lactams [70,71]. Although the presence of PBPs per se does not confer resistance, chromosomal mutations in PBPs may render the bacteria resistant to β-lactams [72-75].

**Figure 15:**
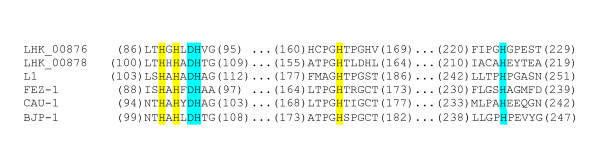
**Multiple alignment of the partial amino acid sequences of the two putative metallo-β-lactamases in *L. hongkongensis *and those of known metallo-β-lactamases showing the conserved zinc-ligand binding sites**. Amino acid residues high-lighted in yellow and blue representing two independent putative zinc-ligand binding sites of class B3 metallo-β-lactamase His116-His118-His196 and Asp120-His121-His263. Numbers in parentheses indicate the corresponding positions in the amino acid sequences. L1, *Stenotrophomonas maltophilia *IID1275 (accession no. CAA52968); FEZ-1, *Legionella gormanii *ATCC33297 (accession no. CAB96921); CAU-1, *Caulobacter vibrioides *DSM 4727 (accession no. CAC87665); BJP-1, *Bradyrhizobium japonicum *USDA 110 (accession no. NP_772870)

Apart from the *ampC *gene (LHK_03028) that encodes the previously characterized class C β-lactamase [76], there are two other putative β-lactamases (LHK_00876 and LHK_00878) observed in the *L. hongkongensis *genome. They are both putative metallo-β-lactamases containing a metallo-β-lactamase superfamily domain which included two zinc ligand-binding sites essential for its hydrolytic function on the β-lactam ring (Figure 15) [77-79]. However, these zinc ligand-binding sites were also present in most proteins of the metallo-β-lactamase superfamily, the function of which is not limited to β-lactam hydrolysis [79-81]. Therefore, *in vitro *experiments are required to confirm the actual function of these two putative metallo-β-lactamases.

#### Multidrug resistance genes

A total of 54 CDSs related to multidrug efflux were identified in *L. hongkongensis *genome (Table 3). The five major families of drug extrusion translocases were all present, including the Major Facilitator Superfamily (MFS) (7 CDSs), Small Multidrug Resistance (SMR) family (2 CDSs), RND family (7 CDSs), Multidrug and Toxic compound Extrusion (MATE) family (2 CDSs), and ATP-Binding Cassette (ABC) superfamily (6 CDSs).

#### Resistance-Nodulation-cell Division (RND) family proteins

For Gram-negative bacteria, the efflux pumps that are associated with most clinically significant resistance to antibiotics are those of the RND family. In this family, three gene loci homologous to *acrRAB-tolC *(LHK_00138, LHK_00140-00142; LHK_02129-02132; LHK_02825-02828) and one gene locus homologous to *acrAD-tolC *(LHK_02929-02931) of *Escherichia coli *were identified in the genome of *L. hongkongensis*. These three AcrRAB-TolC and the AcrAD-TolC multidrug efflux systems shared typical tripartite structure with other multidrug efflux systems in the RND family [82]. AcrB and AcrD are membrane transporter proteins, AcrA is membrane fusion protein and TolC is outer membrane channel protein. *acrR *is a transcription regulator gene located upstream of the *acrAB-tolC *loci. As a multidrug efflux system with broad-substrate spectrum, AcrAB-TolC confers resistance to chloramphenicol, tetracyclines, erythromycin, trimethoprim, β-lactams, and other organic and inorganic antiseptic agents in *E. coli *[83,84]. AcrAD-TolC is less commonly reported compared to AcrAB-TolC system, where AcrD is a close homolog of AcrB. AcrAD-TolC multidrug efflux system is capable of exporting antibiotics of the aminoglycoside class including amikacin, gentamicin, neomycin, kanamycin, tobramycin, and streptomycin in *E. coli *[85,86]. Another putative multidrug efflux system of the RND family identified in the genome of *L. hongkongensis *is homologous to MdtABC-TolC system (LHK_01285, LHK_01286, LHK_01288, LHK_01289). MdtABC-TolC system in *E. coli *confers at least novobiocin and bile salt resistance in the bacterium. A uniqueness of this system is that MdtB and MdtC will form a heterodimer as a membrane efflux component in cooperation with membrane fusion protein MdtA and outer membrane channel protein TolC. [27,87] Moreover, one RND family multidrug efflux system with homology to hydrophobe/amphiphile efflux-1 subfamily was also discovered (LHK_01424-01426).

#### Major Facilitator Superfamily (MFS)

Two loci (LHK_01373-01376; LHK_03132-03134) homologous to emrAB-tolC system of *E. coli *belonging to MFS were found in the genome of *L. hongkongensis*. One of them had an additional transcription regulator *emrR *gene (LHK_01376) in its upstream sequence. EmrAB-TolC system in *E. coli *confers nalidixic acid and other toxic novobiocin substances resistance to bacterium [88]. Moreover, mutation of the *emrR *gene has been shown to lead to over-expression of the EmrAB pump and increased resistance to antimicrobial agents [89]. However, the substrate specificity of these EmrAB-TolC homologs identified in the genome of *L. hongkongensis *is yet to be investigated. There are five other multidrug efflux proteins belonging to MFS (LHK_00743; LHK_01870; LHK_02173; LHK_02539; LHK_02975) in the *L. hongkongensis *genome. One of them (LHK_00743) is a homolog to *mdfA *gene while another (LHK_02975) has high identities to *bcr *gene. *mdfA *encodes an MF-related protein, MdfA, which results in resistance to a diverse group of cationic and zwitterionic lipophilic compounds and antibiotics such as chloramphenicol and erythromycin when over-expressed in *E. coli *[90]. *bcr *gene codes for an efflux protein which is associated with bicyclomycin resistance in *E. coli *[91].

#### Small Multidrug Resistance (SMR) family

Two adjacently located multidrug efflux genes (LHK_01384 and LHK_01385) of the SMR family were identified in the genome of *L. hongkongensis*. They are homologous to *mdtJI *(also named *ydgEF*) genes in *E. coli *which confers resistance to spermidine and, deoxycholate and sodium dodecyl sulfate at low level [92,93]. *mdtJI *have to be co-expressed for functionality and it is suggested that MdtJI may function as a heterodimer or heterooligomer [92-94].

#### Multidrug and Toxic compound Extrusion (MATE) family

Two multidrug efflux genes of the MATE family (LHK_00466 and LHK_02533) were also discovered in the genome of *L. hongkongensis*. One of them (LHK_02533) is a homolog of multidrug efflux protein NorA from *Staphylococcus aureus*, which confers resistance to antibiotics of the quinolone class and various organic compounds [95,96]. Mutation of the *norA *gene in *S. aureus *has resulted in 5- to 30-fold increase in susceptibility to norfloxacin [96].

#### ATP-Binding Cassette (ABC) superfamily

Six CDSs of the ABC transporter family related to multidrug resistance were identified in the *L. hongkongensis *genome. A tripartite multidrug efflux system of the ABC transporter family composed of membrane transporter (LHK_02239), MFP (LHK_02240), and OMP (LHK_02241) was identified in the genome of *L. hongkongensis*. This system of proteins probably functions as a complex with composition resembling to that of RND family. Five other standalone putative ABC transporter genes (LHK_00222; LHK_01967; LHK_02051; LHK_02238; LHK_02949) coding for multidrug efflux proteins were scattered over the *L. hongkongensis *genome. One (LHK_02949) of them possessed homology to *msbA *from *E. coli*, which is responsible for mediating the transport of the lipid A core of LPS to the outer membrane [97,98]. Interestingly, expression of *E. coli *MsbA in *Lactococcus lactis *which lacks LPS has been shown to significantly increase resistance to erythromycin [98].

In addition to these five major families, the *L. hongkongensis *genome also encodes a number of other possible multidrug resistance-related genes. Among these, there are five *marC*-like genes (LHK_01214; LHK_01383; LHK_01934; LHK_02292; LHK_02783), the expression of which was once believed to be associated with multidrug efflux system MarRAB in *E. coli *[99]. However, a recent report has shown that mutation in *marC *did not increase antibiotic susceptibility on *E. coli *[100]. Therefore, the actual function of MarC is still not identified yet. One CDS (LHK_02235) coding for a protein with 75% amino acid identities to putative integral membrane efflux protein of *Yersinia pestis *and possessing an AbgT family domain was also identified in the genome of *L. hongkongensis*. AbgT protein family includes two transporter members, AbgT protein of *E. coli *and MtrF of *N. gonorrhoeae *[101,102]. MtrF, as an inner membrane protein, which enhances the activity of multidrug efflux system MtrCDE of the RND family, conferring higher level of resistance to hydrophobic antibiotics such as penicillin and erythromycin etc. [102,103]. Since no *mtrCDE *gene homologs were found in the genome of *L. hongkongensis*, the role and function of the AbgT family protein in *L. hongkongensis *remains to be elucidated.

#### Miscellaneous resistance genes

Six other CDSs with homologies to other drug resistance genes were identified in the *L. hongkongensis *genome (Table 4). A putative dimethyladenosine transferase, encoded by ksgA gene (LHK_00025) was found. Kasugamycin and streptomycin resistance as a result of mutations in *ksgA *have been documented [104-106]. A *bacA *gene (LHK_02940) encoding putative bacitracin resistance protein BacA was also identified. BacA protein confers bacitracin resistance to *E. coli *by catalyzing the dephosphorylation of undecaprenyl diphosphate (C55-PP) into C55-P, which is important in peptidoglycan synthesis. The conversion of C55-PP into C55-P is normally catalyzed by a specific phosphatase which is inhibited by bacitracin leading to halted peptidoglycan synthesis [107]. The other four CDSs encode putative arsenical-resistance protein (LHK_00913), two camphor resistance proteins CrcB (LHK_01038 and LHK_01039), and chloramphenicol sensitive protein RarD (LHK_01350). Overexpression of CrcB in *E. coli *has been shown to protect the bacteria against chromosome decondensation by camphor [108]. The presence of two *crcB *genes in *L. hongkongensis *genome, but only one copy in the closely related bacterium, *C. violaceum*, and none in *N. gonorrheae *or *N. meningitidis *genomes suggested that this is an important defense mechanism in *L. hongkongensis*. Since the *L. hongkongensis *strain, HLHK9, used for genome sequencing is susceptible to tetracycline (MIC = 0.5 μg/ml), the *tetA *gene previously identified in *L. hongkongensis *strains resistant to tetracycline is not found in the present genome [109]. Recently, class 1 integrons carrying multiple antimicrobial resistance genes were identified in 6.5% of *L. hongkongensis *isolates from aquatic products in Guangzhou city, China [110]. However, such integron is not present in the genome of strain HLHK9.

### Bacteriophages

**Figure 16:**
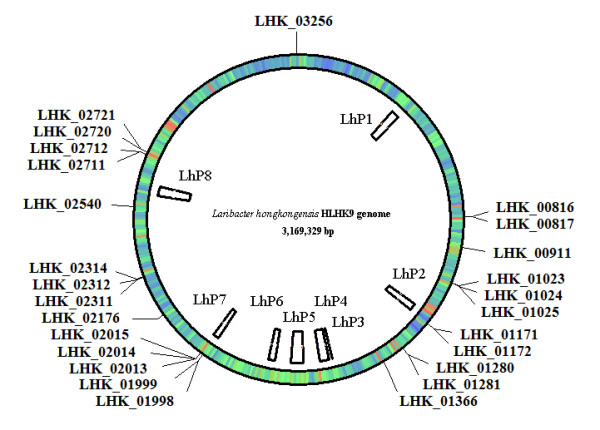
**Position of the LhP prophages and the CDSs coding for transposases in the *L. hongkongensis *genome**. LhP1 to LhP8: *L. hongkongensis *prophages 1 to 8.

The *L. hongkongensis *genome (genome size 3.16 Mbp) contains a total of eight putative prophages named LhP1 to LhP8, the positions of which are shown in Figure 16 and Table 5. This high number of prophages, compared to 3 prophages in *C. violaceum *(genome size 4.75 Mbp) (GenBank accession no. AE016825), 1 to 3 in *N. meningitidis *(genome size 2.14 to 2.27 Mbp) (GenBank accession no. CP000381, FM999788, AM421808, AE002098, AL157959, AM889136, CP001561) and 6 in *N. gonorrhoeae *(genome size 2.15 to 2.23 Mbp) (GenBank accession no. AE004969, CP001050) using the same parameters for prophage prediction by Prophage Finder, suggested that this is an important mechanism for acquisition and exchange of genetic materials in *L. hongkongensis*. While *N. meningitides *and *C. violaceum *cause mainly meningitis and invasive infections respectively that can lead to fatal septicemia, *N. gonorrheae *and *L. hongkongensis *were mainly isolated from human genital and gastrointestinal tract respectively. Interestingly, the presence of apparently high number of prophages also in *N. gonorrhoeae *is in line with our previous observation that horizontal gene transfer was particularly frequent among bacteria residing in human gastrointestinal and probably genital tract [111], suggesting that these anatomical sites may be an excellent incubator for bacterial gene transfer.

**Table 5:** Prophages of *L. hongkongensis *HLHK9

Prophage	Nucleotide Positions	Size (bp)	GC (%)	No. of CDS	Total No. of Phage related CDS	Phage related CDS (No.)
LhP1	356028-387345	31,318	63.07	47	34	P2-like (29), unclassified *Myoviridae *(2), unclassified *Siphoviridae *(2), Mu-like (1)
LhP2	1109928-1136068	26,141	64.81	32	25	Mu-like (10), P2-like (5), lambda-like (3), unclassified phage (3), epsilon15-like (1), unclassified *Myoviridae *(1), unclassified *Podoviridae *(1), unclassified *Siphoviridae *(1)
LhP3	1454673-1465841	11,169	58.70	19	14	BPP-1-like (3), lambda-like (3), epsilon15-like (2), unclassified *Podoviridae *(2), P1-like viruses (1), unclassified *Myoviridae *(1), unclassified *Siphoviridae *(1), unclassifed phage (1)
LhP4	1477589-1511963	34,375	58.78	36	23	BPP-1-like (14), P4-like (4), P2-like (1), P22-like (1), epsilon15-like (1), unclassified *Siphoviridae *(1), unclassified *Myoviridae *(1)
LhP5	1568789-1612785	43,997	59.00	64	32	Mu-like (9), lambda-like (7), unclassified *Podoviridae *(5), unclassified phage (4), unclassified *Myoviridae *(3), unclassified *Siphoviridae *(2), P2-like (1), P22-like (1)
LhP6	1671244-1693161	21,918	62.04	31	25	unclassified *Siphoviridae *(12), lambda-like (5), unclassified phage (3), T1-like (2), unclassified *Myoviridae *(2), unclassified *Podoviridae *(1)
LhP7	1888197-1908188	19,992	55.59	31	18	unclassified phage (7), Mu-like (4), unclassified *Myoviridae *(2), lambda-like (1), P22-like (1), unclassified *Podoviridae *(1), unclassified *Siphoviridae *(1), BPP-1-like (1)
LhP8	2462791-2496581	33,791	63.87	48	37	P2-like (30), *Myoviridae *(2), unclassified *Siphoviridae *(2), unclassified phage (2), Mu-like (1)

#### LhP1

**Figure 17:**
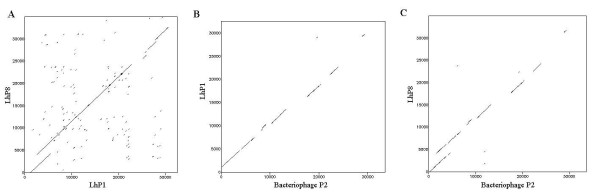
**Dot-plot analysis for LhP1, LhP8 and *E. coli *phage P2**. (A) Dot-plot alignment of LhP8 sequences (vertical axis) versus LhP1 sequences (horizontal axis). (B) Dot-plot alignment of LhP1 sequences (vertical axis) versus *Enterobacteria *phage P2 sequences (horizontal axis). (C) Dot-plot alignment of LhP8 sequences (vertical axis) versus *Enterobacteria *phage P2 sequences (horizontal axis).

Bacteriophage LhP1 is composed of 47 CDSs, accounting for 31,318 bp with G+C content 63.07%, close to the G+C content of the *L. hongkongensis *genome. LhP1 contains 34 phage-related CDSs. Analysis of these CDSs indicated that LhP1 is likely a P2-like phage, as 29 of its 34 phage-related CDSs were most similar to CDSs in P2-like prophages (Figure 17). A P2-like phage typically possesses an icosahedral head with a diameter of about 60 nm, containing a linear double-stranded DNA molecule of about 30-35 kb with cohesive ends and a straight tail with a contractile sheath [112]. Based on their morphology, P2-like phages are classified as members of the *Myoviridae *family (phages with contractile tails) in the order *Caudovirales *(tailed phages) [113]. Other CDSs exhibit similarity to other genes of phages such as Mu-like phages and unclassified phages under *Myoviridae *and *Siphoviridae *(phages with long non-contractile tails).

#### LhP2

Bacteriophage LhP2 is composed of 32 CDSs, accounting for 26,141 bp with G+C content 64.81%. Analysis of its CDSs indicated that LhP2 is likely a Mu-like phage, with 10 of the 25 phage-related CDSs most similar to CDSs in Mu-like phages of *C. violaceum *(CvP1), *Haemophilus influenzae *and *N. meningitidis*. There are also other CDSs similar to other phage genes of lambda- and P2-like phages.

#### LhP3

Bacteriophage LhP3 is the smallest prophage in the *L. hongkongensis *genome. It is composed of 19 CDSs, accounting for 11,169 bp with G+C content 58.70%, lower than that of the host genome (62.35%), reflecting its heterologous origin. Of the 19 CDSs, 14 were phage-related CDSs with similarity to genes of BPP-1-, lambda- and epsilon15-like phages and other unclassified phages, indicating its genetic complexity. Further studies are required if this relatively small prophage is a functional tailed phage.

#### LhP4

Bacteriophage LhP4 is composed of 36 CDSs, accounting for 34,375 bp with G+C content 58.78%, also lower than that of the host genome, indicating its heterologous origin. Of the 23 phage-related CDSs, 14 possessed similarity to genes of *Bordetella *phage BPP-1. Other phage related genes resemble those of P4-, P2-, P22- and episolon15-like phages and unclassified phages of *Siphoviridae *and *Myoviridae*.

#### LhP5

Bacteriophage LhP5 is the largest prophage identified in the *L. hongkongensis *genome. Composed of 64 CDSs, it accounts for 43,997 bp with G+C content 59%, lower than that of the host genome. Of the 32 phage-related CDSs, 9 possessed homologies to genes of Mu-like phages, 7 even possessed homologies to genes of lambda-like phages. The other phage-related CDSs are most closely related to those of various phages including those belonging to *Podoviridae *(phages with short tails), *Myoriviridae *and *Siphoviridae*.

#### LhP6

Bacteriophage LhP6 is composed of 31 CDSs, accounting for 21,918 bp with G+C content of 62.04%. The 25 phage-related CDSs exhibit similarity to phage genes of *Bordetella bronchiseptica *and *Bordetella avium*. Of these 25 CDSs, 12 possessed homologies to genes of unclassified phages belonging to *Siphoviridae *and 5 to lambda-like phages.

#### LhP7

Bacteriophage LhP7 is composed of 31 CDSs, accounting for 19,992 bp with the lowest G+C content of 55.59% among the eight prophages, suggesting a heterologous origin. Of the 18 phage-related CDSs, 4 exhibits similarity to phage genes of *N. meningitidis*, *Burkholderia*, and *C. violaceum *genes of Mu-like phages, and others to those of unclassified phages, lambda-, P22-, and BPP-1-like phages.

#### LhP8

Similar to LhP1, bacteriophage LhP8 is also a P2-like phage (Figure 17). It is composed of 48 CDSs, accounting for 33,791 bp with G+C content of 63.87%, similar to that of the host genome. It contains the highest number of phage-related CDSs (n = 37) among the eight phages. Of the 37 phage-related CDSs, 30 were most similar to genes of P2-like phages and others to phages of *Myoviridae*, *Siphoviridae *and Mu-like phages. In fact, LhP1 and LhP8 are highly similar with the exception of a few CDSs, with most of their CDSs exhibiting similarity to phage proteins found in other gram-negative bacteria including *Salmonella*, *Burkholderia*, *Yersinia*, and *Shigella *species. Their gene organizations are also highly similar to P2 phage (Table 6) (Figure 17).

**Table 6:** CDSs of LhP1 and LhP8 from the *L. hongkongensis *HLHK9, and comparison of genome structures of LhP1 (reverse complement), LhP8 and *E. coli *P2 phage.

P2	LhP1	LhP8	Function
Q	LHK_00420	LHK_02579	Capsid portal protein
P	LHK_00419	LHK_02580/LHK_02582	Large terminase subunit
O	LHK_00418	LHK_02581/LHK_02583	Capsid scaffold
N	LHK_00417	LHK_02584	Major capsid precursor
M	LHK_00416	LHK_02585	Small terminase subunit
L	LHK_00415	LHK_02586	Capsid completion
X	LHK_00414	LHK_02587	Tail
Y	-	-	Lysis - holin
K	-	-	Lysis - endolysin
-	LHK_00413	LHK_02588	Phage-related transmembrane protein
-	LHK_00412	LHK_02589	Hypothetical protein
-	LHK_00411	LHK_02590	Putative phage-related protein (hydrolase)
lysA	-	-	Timing of lysis
lysB	LHK_00410	LHK_02591	Timing of lysis
lysC	-	-	Regulation of lysis
-	LHK_00409	LHK_02592	Hypothetical protein
R	LHK_00408	LHK_02593	Tail completion
S	LHK_00407	LHK_02594	Tail completion
V	LHK_00406/LHK_00405	LHK_02595	Baseplate assembly
W	LHK_00404	LHK_02596	Baseplate assembly
J	LHK_00403	LHK_02597	Baseplate assembly
I	LHK_00402	LHK_02598	Baseplate assembly
H	LHK_00401	LHK_02599	Tail fiber
-	LHK_00400	LHK_02600	Hypothetical protein
-	LHK_00399	LHK_02601	Mu-like prophage protein Com
-	LHK_00398	LHK_02602	DNA adenine methylase
-	LHK_00397	LHK_02603	Hypothetical protein
G	-	-	Tail fiber assembly
Z/fun	-	-	Blocks phage T5
FI	LHK_00396	LHK_02604	Tail sheath
FII	LHK_00395	LHK_02605	Tail tube
E+E'	LHK_00394	LHK_02606	Tail
E	LHK_00393	LHK_02607	Tail
T	LHK_00392	LHK_02608	Tail
U	LHK_00391	LHK_02609	Tail
D	LHK_00390	LHK_02610	Tail
-	LHK_00389	-	Hypothetical protein
-	LHK_00388	-	Hypothetical protein
-	LHK_00387	-	Hypothetical protein
-	LHK_00386	-	Hypothetical protein
-	LHK_00385	-	Hypothetical protein
-	-	LHK_02611	Anthranilate synthase component I
-	-	LHK_02612	Hypothetical protein
-	-	LHK_02613	Hypothetical protein
-	-	LHK_02614	Hypothetical protein
-	LHK_00384	LHK_02615	Hypothetical protein
Ogr	LHK_00383	LHK_02616	Late promoter activator
-	LHK_00382	LHK_02617	Hypothetical protein
-	LHK_00381	-	Hypothetical protein
-	LHK_00380	LHK_02618	Hypothetical protein
-	-	LHK_02619	Hypothetical protein
-	LHK_00379	LHK_02620	Cro/CI family transcriptional regulator
-	LHK_00378	LHK_02621	Hypothetical protein
Int	-	-	Integrase
C	-	-	Immunity repressor
Cox	-	-	Inhibits integration
B	-	-	DNA replication
A	LHK_00377	LHK_02622	DNA replication
-	LHK_00376	-	Hypothetical protein
-	LHK_00375	-	Hypothetical protein
-	-	LHK_02623	Hypothetical protein
-	-	LHK_02624	DNA binding protein, excisionase family
tin	-	-	Blocks growth of T-even phages
old	-	-	Blocks growth of phage lambda
-	LHK_00374	LHK_02625	Integrase

#### Remnant phages

Among the eight putative prophages, LhP1 and LhP8 are most likely to represent intact prophages, while the remaining six prophages encode a diversity of prophage elements of phage-related structural and non-structural proteins. In addition to these putative prophages, 17 other phage-related CDSs were found scattered in the *L. hongkongensis *genome. However, these CDSs are either not flanked by other phage-related genes or that the region of these phage-related gene clusters was too short for confident prediction as prophages. Further studies are required to ascertain if the present putative prophages and phage-related gene clusters are intact or remnant phages.

### Transposases and insertion sequences

There are 26 CDSs coding for transposases in the *L. hongkongensis *genome (Table 7). Fourteen of these 26 transposases possessed homologies to transposases of IS3 family, nine to those of IS5 family and three to those of IS481 family. The presence of transposases of IS481 family is unique in *L. hongkongensis*, as they are absent in other members of the *Neisseriaceae *family such as the pathogenic *Neisseria *species and *C. violaceum *[114]. The transposases of *L. hongkongensis *are most closely related to those of other members of β-proteobacteria, especially of the order *Burkholderiales*, with seven most closely related to those of *Comamonas testosteroni*, seven to those of *Janthinobacterium *sp., and four to those of *Polaromonas *sp. However, only two pairs of these transposases carry short imperfect inverted repeats at their ends that form insertion sequences most closely related to the IS3 family. Other transposases are likely remnant insertion sequences and lack associated inverted repeats. The first insertion sequence, of 1,183 bp, contains two ORFs, LHK_01280 (ORFb) and LHK_01281 (ORFa), with 38-bp inverted repeats with six mismatches. The second insertion sequence is relatively short in length, with 603 bp containing two ORFs, LHK_02311 and LHK_02312 (ORFa) and 50-bp inverted repeats with ten mismatches. The G+C content of both putative insertion sequences are lower (57.4% and 54.89% respectively) than that of the *L. hongkongensis *genome, suggestive of heterologous origin.

**Table 7:** Transposases identified in the genome of *L. hongkongensis *HLHK9

CDS	IS name	IS family	IS group	Origin	Identity (%)	E-value	Size (bp)
LHK_00816	ISCte3	IS3	IS407	*Comamonas testosteroni*	79.76	2e-38	294
LHK_00817	ISCte3	IS3	IS407	*Comamonas testosteroni*	77.01	9e-36	264
LHK_00911	ISAisp3	IS481	-	*Acidovorax *sp.	62.5	7e-54	588
LHK_01023	ISJsp2	IS5	IS903	*Janthinobacterium *sp.	47.92	6e-50	822
LHK_01024	ISPosp5	IS3	IS3	*Polaromonas *sp.	71.21	2e-24	339
LHK_01025	ISPosp5	IS3	IS3	*Polaromonas *sp.	65.98	1e-33	336
LHK_01171	ISPosp5	IS3	IS3	*Polaromonas *sp.	71.21	2e-24	342
LHK_01172	ISPosp5	IS3	IS3	*Polaromonas *sp.	65.98	2e-33	336
LHK_01280	ISKpn10	IS3	IS407	*Klebsiella pneumoniae*	68.29	2e-50	360
LHK_01281	ISKpn10	IS3	IS407	*Klebsiella pneumoniae*	84.09	2e-39	267
LHK_01366	ISJsp2	IS5	IS903	*Janthinobacterium *sp.	60.68	4e-36	744
LHK_01998	ISJsp2	IS5	IS903	*Janthinobacterium *sp.	75.86	2e-09	186
LHK_01999	ISPpa4	IS5	IS903	*Paracoccus pantotrophus*	69	2e-17	219
LHK_02013	ISRme14	IS481	-	*Ralstonia metallidurans*	59.46	2e-22	249
LHK_02014	ISAisp3	IS481	-	*Acidovorax *sp.	71.26	8e-27	276
LHK_02015	ISJsp2	IS5	IS903	*Janthinobacterium *sp.	60.53	7e-35	540
LHK_02176	ISJsp2	IS5	IS903	*Janthinobacterium *sp.	58.02	7e-23	273
LHK_02311	ISCte3	IS3	IS407	*Comamonas testosteroni*	88.89	6e-09	141
LHK_02312	ISCte3	IS3	IS407	*Comamonas testosteroni*	78.05	8e-14	126
LHK_02314	ISCte3	IS3	IS407	*Comamonas testosteroni*	96	3e-20	399
LHK_02540	ISJsp2	IS5	IS903	*Janthinobacterium *sp.	59.54	9e-40	648
LHK_02711	IS476	IS3	IS407	*Xanthomonas campestris *pv. *vesicatoria *81-23 race 2	63.64	7e-42	387
LHK_02712	IS1421	IS5	IS427	*Ralstonia solanacearum*	57.38	8e-33	357
LHK_02720	ISCte3	IS3	IS407	*Comamonas testosteroni*	72.73	4e-14	627
LHK_02721	ISCte3	IS3	IS407	*Comamonas testosteroni*	77.01	4e-12	264
LHK_03256	ISJsp2	IS5	IS903	*Janthinobacterium *sp.	62.79	3e-27	477

## Conclusions

The *L. hongkongensis *genome possessed genes and gene cassettes for acid and bile resistance, colonization of the intestinal mucosa, evasion of host defense and cytotoxicity and invasion. In addition, a broad variety of antibiotic resistance or multidrug resistance genes, a high number of prophages, together with other phage-related CDSs and CDSs coding for transposases, were also identified.

## Methods

CDSs identified in the *L. hongkongensis *genome were annotated as described in our previous publication and classified functionally according to the Clusters of Orthologous Groups (COG) methodology [10]. CDSs belonging to COG clusters potentially associated with virulence (such as intracellular trafficking, secretion and vesicular transport) were selected for further examination, whereas those associated with housekeeping functions (such as chromatin structure and dynamics) were removed. The CDSs were then examined by comparison with the latest release of the reference Virulence Factor Database (VFDB) [115] and keyword searching using the following words and their variants: virulence, toxin, hemolysin/hemolysis, pathogenicity, adherence, invasion, secretion, phagocytosis, phase variation, stress, iron uptake, siderophore, resistance, efflux pump, damaging and regulation. For drug resistance, CDSs that were classified to COG V (defense mechanisms), COG Q (secondary metabolites biosynthesis, transport and catabolism), and COQ M (cell wall/membrane/envelope biogenesis) were manually annotated for identification of antibiotic resistance-related genes. CDSs from other COGs were searched for additional genes using keywords: resistance antibiotic, efflux, multi etc. Prophages were identified by Prophage finder http://bioinformatics.uwp.edu/~phage/ searches [116]. The genome was run under the parameters with an e-value of 0.01, hits per prophage of 7, and hit spacing of 5000. Transposases were identified by performing BlastP analyses for all CDSs identified in the genome of *L. hongkongensis *HLHK9 against the ISfinder database http://www-is.biotoul.fr/is.html[117] and inverted repeats by einverted (EMBOSS package) [118]. Manual confirmation of the assigned function was performed by sequence similarity search using BLAST against the NCBI nr database, and assisted by conserved domain search (CD-search), identification of signature sequence motifs and sequence analysis using InterProScan. Localization patterns of putative virulence factors were predicted using PSORTb where appropriate [119].

## List of abbreviations

ABC: ATP-Binding Cassette; ATP: Adenosine triphosphate; BLAST: Basic Local Alignment Search Tool; bp: Base pair; C55-P: Undecaprenyl pyrophosphate; C55-PP: Undecaprenyl diphosphate; CD14: Cluster of differentiation 14; CDS(s): Coding sequence(s); COG(s): Clusters of orthologous group(s); CvP: *Chromobacterium violaceum *phage; DAEC: Diffusely adherent *Escherichia coli*; DNA: Deoxyribonucleic acid; dTDP: Deoxythymidine diphosphate; EMBOSS: European Molecular Biology Open Software Suite; ETEC: Enterotoxigenic *Escherichia coli*; IS: Insertion sequence; Kdo: Keto-deoxyoctulonate; LhP: *Laribacter hongkongensis *prophage; LPS: Lipopolysaccharide; MATE: Multidrug and Toxic compound Extrusion; Mbp: Mega base pairs; MF: Major facilitator; MFP: Membrane fusion protein; MFS: Major Facilitator Superfamily; MIC: Minimum inhibitory concentration; OMP: Outer membrane (channel) protein; OMPLA: Outer membrane phospholipase A; ORF(s): Open reading frame(s); PBP(s): Penicillin-binding protein(s); RND: Resistance nodulation division; RTX: Repeats in toxin; SMR: Small Multidrug Resistance family.

## Competing interests

The authors declare that they have no competing interests.

## Authors' contributions

PCYW, KYY and SKPL designed and supervised the study. GKMW, AKLT JLLT, and RYYF annotated the genome. HT performed bioinformatics analysis. SKPL, GKMW, AKLT and PCYW drafted the manuscript. All authors corrected the manuscript. All authors read and approved the final manuscript.
